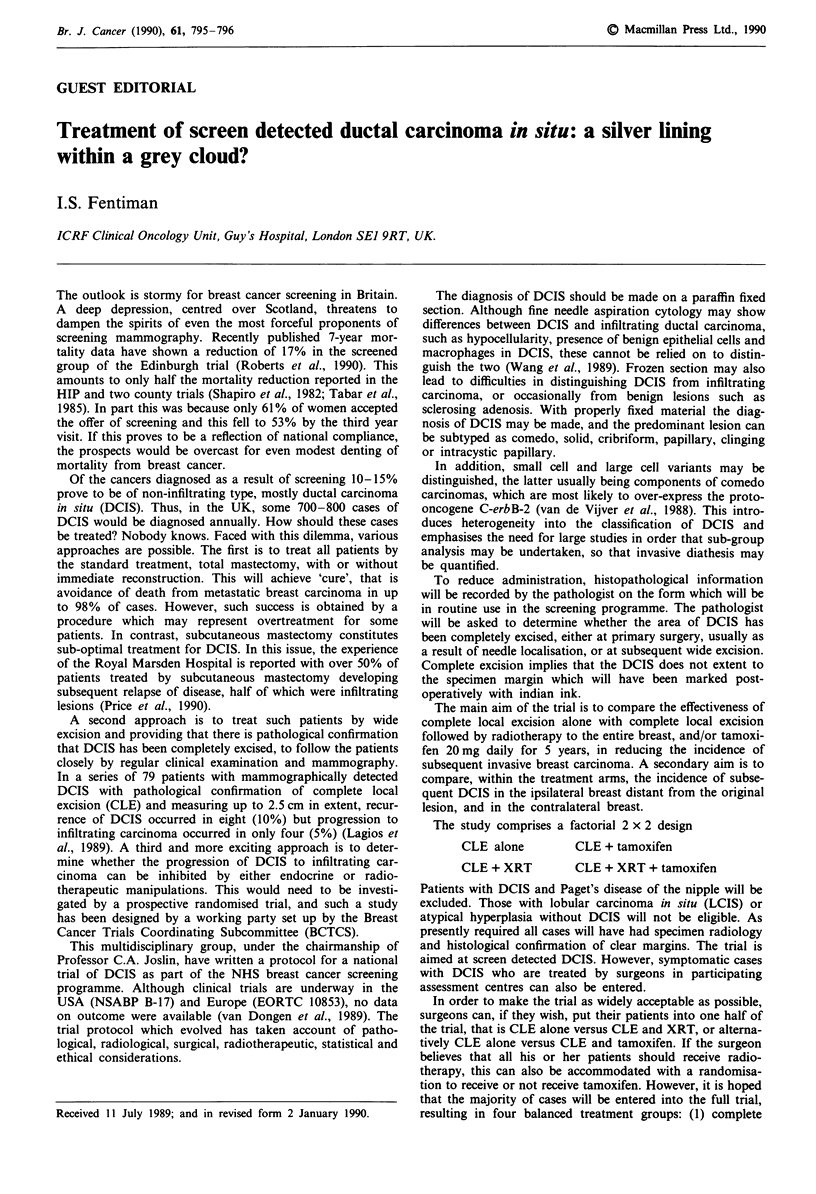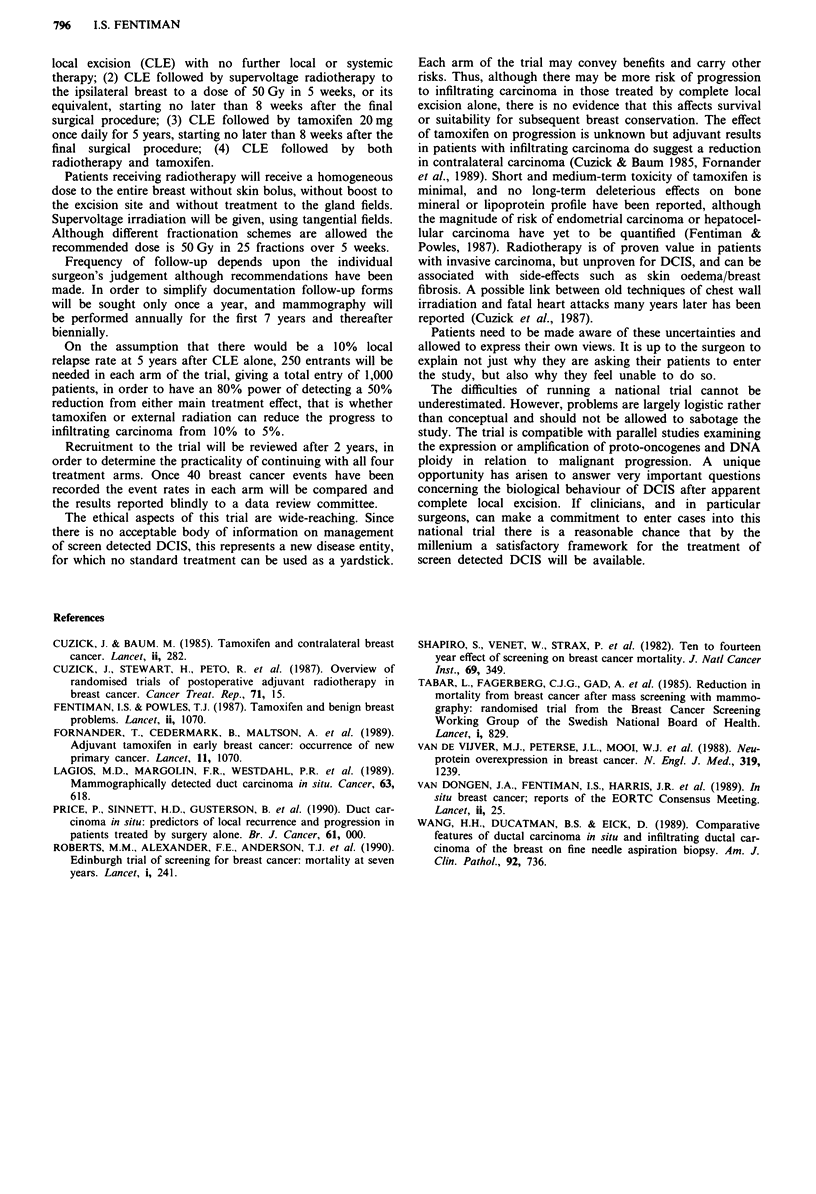# Treatment of screen detected ductal carcinoma in situ: a silver lining within a grey cloud?

**DOI:** 10.1038/bjc.1990.179

**Published:** 1990-06

**Authors:** I. S. Fentiman


Br. J. Cancer (1990), 61, 795-796 © Macmillan Press Ltd., 1990

GUEST EDITORIAL

### Treatment of screen detected ductal carcinoma *in situ*: a silver lining within a grey cloud?

I.S. Fentiman

*ICRF Clinical Oncology Unit, Guy's Hospital, London SE1 9RT, UK.*

The outlook is stormy for breast cancer screening in Britain. A deep depression, centred over Scotland, threatens to dampen the spirits of even the most forceful proponents of screening mammography. Recently published 7-year mortality data have shown a reduction of 17% in the screened group of the Edinburgh trial (Roberts *et al.*, 1990). This amounts to only half the mortality reduction reported in the HIP and two county trials (Shapiro *et al.*, 1982; Tabar *et al.*, 1985). In part this was because only 61% of women accepted the offer of screening and this fell to 53% by the third year visit. If this proves to be a reflection of national compliance, the prospects would be overcast for even modest denting of mortality from breast cancer.

Of the cancers diagnosed as a result of screening 10-15% prove to be of non-infiltrating type, mostly ductal carcinoma *in situ* (DCIS). Thus, in the UK, some 700-800 cases of DCIS would be diagnosed annually. How should these cases be treated? Nobody knows. Faced with this dilemma, various approaches are possible. The first is to treat all patients by the standard treatment, total mastectomy, with or without immediate reconstruction. This will achieve 'cure', that is avoidance of death from metastatic breast carcinoma in up to 98% of cases. However, such success is obtained by a procedure which may represent overtreatment for some patients. In contrast, subcutaneous mastectomy constitutes sub-optimal treatment for DCIS. In this issue, the experience of the Royal Marsden Hospital is reported with over 50% of patients treated by subcutaneous mastectomy developing subsequent relapse of disease, half of which were infiltrating lesions (Price *et al.*, 1990).

A second approach is to treat such patients by wide excision and providing that there is pathological confirmation that DCIS has been completely excised, to follow the patients closely by regular clinical examination and mammography. In a series of 79 patients with mammographically detected DCIS with pathological confirmation of complete local excision (CLE) and measuring up to 2.5 cm in extent, recurrence of DCIS occurred in eight (10%) but progression to infiltrating carcinoma occurred in only four (5%) (Lagios *et al.*, 1989). A third and more exciting approach is to determine whether the progression of DCIS to infiltrating carcinoma can be inhibited by either endocrine or radiotherapeutic manipulations. This would need to be investigated by a prospective randomised trial, and such a study has been designed by a working party set up by the Breast Cancer Trials Coordinating Subcommittee (BCTCS).

This multidisciplinary group, under the chairmanship of Professor C.A. Joslin, have written a protocol for a national trial of DCIS as part of the NHS breast cancer screening programme. Although clinical trials are underway in the USA (NSABP B-17) and Europe (EORTC 10853), no data on outcome were available (van Dongen *et al.*, 1989). The trial protocol which evolved has taken account of pathological, radiological, surgical, radiotherapeutic, statistical and ethical considerations.

Received 11 July 1989; and in revised form 2 January 1990.

The diagnosis of DCIS should be made on a paraffin fixed section. Although fine needle aspiration cytology may show differences between DCIS and infiltrating ductal carcinoma, such as hypocellularity, presence of benign epithelial cells and macrophages in DCIS, these cannot be relied on to distinguish the two (Wang *et al.*, 1989). Frozen section may also lead to difficulties in distinguishing DCIS from infiltrating carcinoma, or occasionally from benign lesions such as sclerosing adenosis. With properly fixed material the diagnosis of DCIS may be made, and the predominant lesion can be subtyped as comedo, solid, cribriform, papillary, clinging or intracystic papillary.

In addition, small cell and large cell variants may be distinguished, the latter usually being components of comedo carcinomas, which are most likely to over-express the proto-oncogene C-*erb*B-2 (van de Vijver *et al.*, 1988). This introduces heterogeneity into the classification of DCIS and emphasises the need for large studies in order that sub-group analysis may be undertaken, so that invasive diathesis may be quantified.

To reduce administration, histopathological information will be recorded by the pathologist on the form which will be in routine use in the screening programme. The pathologist will be asked to determine whether the area of DCIS has been completely excised, either at primary surgery, usually as a result of needle localisation, or at subsequent wide excision. Complete excision implies that the DCIS does not extent to the specimen margin which will have been marked post-operatively with indian ink.

The main aim of the trial is to compare the effectiveness of complete local excision alone with complete local excision followed by radiotherapy to the entire breast, and/or tamoxifen 20 mg daily for 5 years, in reducing the incidence of subsequent invasive breast carcinoma. A secondary aim is to compare, within the treatment arms, the incidence of subsequent DCIS in the ipsilateral breast distant from the original lesion, and in the contralateral breast.

The study comprises a factorial 2 × 2 design

| | |
|---|---|
| CLE alone | CLE + tamoxifen |
| CLE + XRT | CLE + XRT + tamoxifen |

Patients with DCIS and Paget's disease of the nipple will be excluded. Those with lobular carcinoma *in situ* (LCIS) or atypical hyperplasia without DCIS will not be eligible. As presently required all cases will have had specimen radiology and histological confirmation of clear margins. The trial is aimed at screen detected DCIS. However, symptomatic cases with DCIS who are treated by surgeons in participating assessment centres can also be entered.

In order to make the trial as widely acceptable as possible, surgeons can, if they wish, put their patients into one half of the trial, that is CLE alone versus CLE and XRT, or alternatively CLE alone versus CLE and tamoxifen. If the surgeon believes that all his or her patients should receive radiotherapy, this can also be accommodated with a randomisation to receive or not receive tamoxifen. However, it is hoped that the majority of cases will be entered into the full trial, resulting in four balanced treatment groups: (1) complete local excision (CLE) with no further local or systemic therapy; (2) CLE followed by supervoltage radiotherapy to the ipsilateral breast to a dose of 50 Gy in 5 weeks, or its equivalent, starting no later than 8 weeks after the final surgical procedure; (3) CLE followed by tamoxifen 20 mg once daily for 5 years, starting no later than 8 weeks after the final surgical procedure; (4) CLE followed by both radiotherapy and tamoxifen.

Patients receiving radiotherapy will receive a homogeneous dose to the entire breast without skin bolus, without boost to the excision site and without treatment to the gland fields. Supervoltage irradiation will be given, using tangential fields. Although different fractionation schemes are allowed the recommended dose is 50 Gy in 25 fractions over 5 weeks.

Frequency of follow-up depends upon the individual surgeon's judgement although recommendations have been made. In order to simplify documentation follow-up forms will be sought only once a year, and mammography will be performed annually for the first 7 years and thereafter biennially.

On the assumption that there would be a 10% local relapse rate at 5 years after CLE alone, 250 entrants will be needed in each arm of the trial, giving a total entry of 1,000 patients, in order to have an 80% power of detecting a 50% reduction from either main treatment effect, that is whether tamoxifen or external radiation can reduce the progress to infiltrating carcinoma from 10% to 5%.

Recruitment to the trial will be reviewed after 2 years, in order to determine the practicality of continuing with all four treatment arms. Once 40 breast cancer events have been recorded the event rates in each arm will be compared and the results reported blindly to a data review committee.

The ethical aspects of this trial are wide-reaching. Since there is no acceptable body of information on management of screen detected DCIS, this represents a new disease entity, for which no standard treatment can be used as a yardstick. Each arm of the trial may convey benefits and carry other risks. Thus, although there may be more risk of progression to infiltrating carcinoma in those treated by complete local excision alone, there is no evidence that this affects survival or suitability for subsequent breast conservation. The effect of tamoxifen on progression is unknown but adjuvant results in patients with infiltrating carcinoma do suggest a reduction in contralateral carcinoma (Cuzick & Baum 1985, Fornander *et al.*, 1989). Short and medium-term toxicity of tamoxifen is minimal, and no long-term deleterious effects on bone mineral or lipoprotein profile have been reported, although the magnitude of risk of endometrial carcinoma or hepatocellular carcinoma have yet to be quantified (Fentiman & Powles, 1987). Radiotherapy is of proven value in patients with invasive carcinoma, but unproven for DCIS, and can be associated with side-effects such as skin oedema/breast fibrosis. A possible link between old techniques of chest wall irradiation and fatal heart attacks many years later has been reported (Cuzick *et al.*, 1987).

Patients need to be made aware of these uncertainties and allowed to express their own views. It is up to the surgeon to explain not just why they are asking their patients to enter the study, but also why they feel unable to do so.

The difficulties of running a national trial cannot be underestimated. However, problems are largely logistic rather than conceptual and should not be allowed to sabotage the study. The trial is compatible with parallel studies examining the expression or amplification of proto-oncogenes and DNA ploidy in relation to malignant progression. A unique opportunity has arisen to answer very important questions concerning the biological behaviour of DCIS after apparent complete local excision. If clinicians, and in particular surgeons, can make a commitment to enter cases into this national trial there is a reasonable chance that by the millenium a satisfactory framework for the treatment of screen detected DCIS will be available.

### References

CUZICK, J. & BAUM. M. (1985). Tamoxifen and contralateral breast cancer. *Lancet*, **ii**, 282.

CUZICK, J., STEWART, H., PETO, R. *et al.* (1987). Overview of randomised trials of postoperative adjuvant radiotherapy in breast cancer. *Cancer Treat. Rep.*, **71**, 15.

FENTIMAN, I.S. & POWLES, T.J. (1987). Tamoxifen and benign breast problems. *Lancet*, **ii**, 1070.

FORNANDER, T., CEDERMARK, B., MALTSON, A. *et al.* (1989). Adjuvant tamoxifen in early breast cancer: occurrence of new primary cancer. *Lancet*, **11**, 1070.

LAGIOS, M.D., MARGOLIN, F.R., WESTDAHL, P.R. *et al.* (1989). Mammographically detected duct carcinoma *in situ*. *Cancer*, **63**, 618.

PRICE, P., SINNETT, H.D., GUSTERSON, B. *et al.* (1990). Duct carcinoma *in situ*: predictors of local recurrence and progression in patients treated by surgery alone. *Br. J. Cancer*, **61**, 000.

ROBERTS, M.M., ALEXANDER, F.E., ANDERSON, T.J. *et al.* (1990). Edinburgh trial of screening for breast cancer: mortality at seven years. *Lancet*, **i**, 241.

SHAPIRO, S., VENET, W., STRAX, P. *et al.* (1982). Ten to fourteen year effect of screening on breast cancer mortality. *J. Natl Cancer Inst.*, **69**, 349.

TABAR, L., FAGERBERG, C.J.G., GAD, A. *et al.* (1985). Reduction in mortality from breast cancer after mass screening with mammography: randomised trial from the Breast Cancer Screening Working Group of the Swedish National Board of Health. *Lancet*, **i**, 829.

VAN DE VIJVER, M.J., PETERSE, J.L., MOOI, W.J. *et al.* (1988). *Neu*-protein overexpression in breast cancer. *N. Engl. J. Med.*, **319**, 1239.

VAN DONGEN, J.A., FENTIMAN, I.S., HARRIS, J.R. *et al.* (1989). *In situ* breast cancer; reports of the EORTC Consensus Meeting. *Lancet*, **ii**, 25.

WANG, H.H., DUCATMAN, B.S. & EICK, D. (1989). Comparative features of ductal carcinoma *in situ* and infiltrating ductal carcinoma of the breast on fine needle aspiration biopsy. *Am. J. Clin. Pathol.*, **92**, 736.